# Indirect 3D Bioprinting of a Robust Trilobular Hepatic Construct with Decellularized Liver Matrix Hydrogel

**DOI:** 10.3390/bioengineering9110603

**Published:** 2022-10-22

**Authors:** Vamakshi Khati, Johannes Artturi Turkki, Harisha Ramachandraiah, Falguni Pati, Giulia Gaudenzi, Aman Russom

**Affiliations:** 1 Science for Life Laboratory, Division of Nanobiotechnology, Department of Protein Science, KTH Royal Institute of Technology, 17165 Solna, Sweden; 2 Faculty of Medicine and Health Technology, Tampere University, 33100 Tampere, Finland; 3 Biopromic AB, 17165 Solna, Sweden; 4 Department of Biomedical Engineering, Indian Institute of Technology Hyderabad, Kandi 502285, India; 5 Department of Global Public Health, Karolinska Institute, 17165 Solna, Sweden; 6 AIMES—Center for the Advancement of Integrated Medical and Engineering Sciences, Karolinska Institute and KTH Royal Institute of Technology, 11428 Stockholm, Sweden

**Keywords:** sacrificial scaffold, liver lobule, robust structure, decellularized liver extracellular matrix, indirect 3D bioprinting, co-culture

## Abstract

The liver exhibits complex geometrical morphologies of hepatic cells arranged in a hexagonal lobule with an extracellular matrix (ECM) organized in a specific pattern on a multi-scale level. Previous studies have utilized 3D bioprinting and microfluidic perfusion systems with various biomaterials to develop lobule-like constructs. However, they all lack anatomical relevance with weak control over the size and shape of the fabricated structures. Moreover, most biomaterials lack liver-specific ECM components partially or entirely, which might limit their biomimetic mechanical properties and biological functions. Here, we report 3D bioprinting of a sacrificial PVA framework to impart its trilobular hepatic structure to the decellularized liver extracellular matrix (dLM) hydrogel with polyethylene glycol-based crosslinker and tyrosinase to fabricate a robust multi-scale 3D liver construct. The 3D trilobular construct exhibits higher crosslinking, viscosity (182.7 ± 1.6 Pa·s), and storage modulus (2554 ± 82.1 Pa) than non-crosslinked dLM. The co-culture of HepG_2_ liver cells and NIH 3T3 fibroblast cells exhibited the influence of fibroblasts on liver-specific activity over time (7 days) to show higher viability (90–91.5%), albumin secretion, and increasing activity of four liver-specific genes as compared to the HepG_2_ monoculture. This technique offers high lumen patency for the perfusion of media to fabricate a densely populated scaled-up liver model, which can also be extended to other tissue types with different biomaterials and multiple cells to support the creation of a large functional complex tissue.

## 1. Introduction

Three-dimensional (3D) printing technology is firmly established for complex tailor-made models with diverse materials [1]. In tissue engineering, 3D bioprinting is inching closer to mimicking the tissue-specific microvasculature with multi-component bioinks to fabricate in vitro models for disease modeling, drug screening, and organ replacement [2,3,4,5]. It permits the programmed assembly of different types of cells with multiple bioinks, ranging from high-temperature biopolymers to soft hydrogels [6,7]. With the plethora of functional biomaterials available today, only a few can be utilized as bioink for 3D bioprinting due to their limitations of weak mechanical strength and crosslinking dynamics [8,9,10]. As a result, a very narrow choice of bioinks is available, thereby hindering the development of complex 3D scaffolds. This has prompted the development of different approaches to widen the application of multiple biomaterials in generating a suitable construct without compromising material properties or resolution [11,12,13,14].

Recently, a new method of bioprinting with sacrificial scaffolds, known as Indirect bioprinting, has emerged in which a cytocompatible sacrificial framework temporarily supports the formation of a desirable scaffold with the selected biomaterial incorporated with cells [15,16,17]. Indirect bioprinting renders a high degree of control over the internal and external architecture of the 3D scaffold with a versatile range of biomaterials. It provides a high degree of versatility in creating complex hydrogel scaffolds with various biomaterials and structures, thus enabling clinical success with complex organs such as the liver, heart or spleen [18]. This technique has been utilized previously to develop soft tissue constructs with the help of several support materials such as gelatin for nerve guide conduits [19] and mesoscale pore networks [14], pluronic F127 for vascular grafts [20], poloxamer 407 for the vascular network [21], agarose for mammary ducts such as microchannels [22], and PVA for multiple anatomically shaped tissues [23] or microfluidic channels [24]. Usually, sacrificial scaffolds can be removed by dissolving in an aqueous media [23,25]. They can also be extracted physically [26,27,28] or with temperature changes for the sol-gel transition [4,5,29]. As noted, the biomaterials used in these studies either did not entirely contain any ECM component or did not fully mimic the ideal ratios of a variety of bioactive proteins specific to the target tissue. Furthermore, the most common sacrificial materials used in these studies were pluronic 127 and gelatin because of their shear-thinning behavior and high viscosity. These support materials have good shape fidelity post-printing. However, they have mainly been used to develop in vitro vascular microchannels, and limited progress has been made toward a complex heterogeneous tissue microenvironment [29,30]. FDA-approved cost-effective Polyvinyl alcohol (PVA) has also been used as a sacrificial support material to create multiple anatomically shaped 3D architectures combined with a variety of biomaterials [31,32,33,34]. However, these studies were primarily focused on micro-channel networks or organ-on-chip systems. They did not create multiple anatomically shaped 3D architectures targeting any morphologically complex soft tissue such as the liver, kidney, or heart. Thus, integrating sacrificial organ-specific systems with biomimetic ECM hydrogel would be the quintessential next step.

Mimicking the biological substrates to reproduce the complex 3D features in the liver or similar tissues to a larger geometrical scale is highly desirable for the multi-scale tissue engineering [35]. It is well known that the liver is a highly evolved organ involved in multiple body functions with a very intricate 3D microenvironment of hepatocytes and other liver cells arranged in lobules. The hepatic lobule is hexagonal in shape, with a central vein passing through the center, and it is considered the basic functional unit of a liver [36,37,38,39]. Vascularization is a prerequisite for the clinical translation of any tissue model. Mimicking highly vascularized organs like the liver needs advanced bioprinting techniques [40], such as indirect bioprinting, to fabricate thick liver models multi-scaled in the z-axis to mimic the liver-specific extracellular matrix (ECM) of the native liver [41]. This could be achieved using the decellularized liver extracellular matrix (dLM) that fully replicates the native features of the natural ECM [7], as we have observed in our previous work to develop a mechanically robust bioink with dLM crosslinked with succinimidyl valerate-polyethylene glycol-succinimidyl valerate (xPEGx, x: succinimidyl valerate), gelatin, and mushroom tyrosinase. However, multi-scaling liver tissue with dLM is challenging due to its inherent weak mechanical property [42]. Thus, further crosslinking of dLM to enhance its shape fidelity along with a support structure would be ideal for fabricating a 3D structure with an intricate internal architecture [7,43].

**Figure 1:**
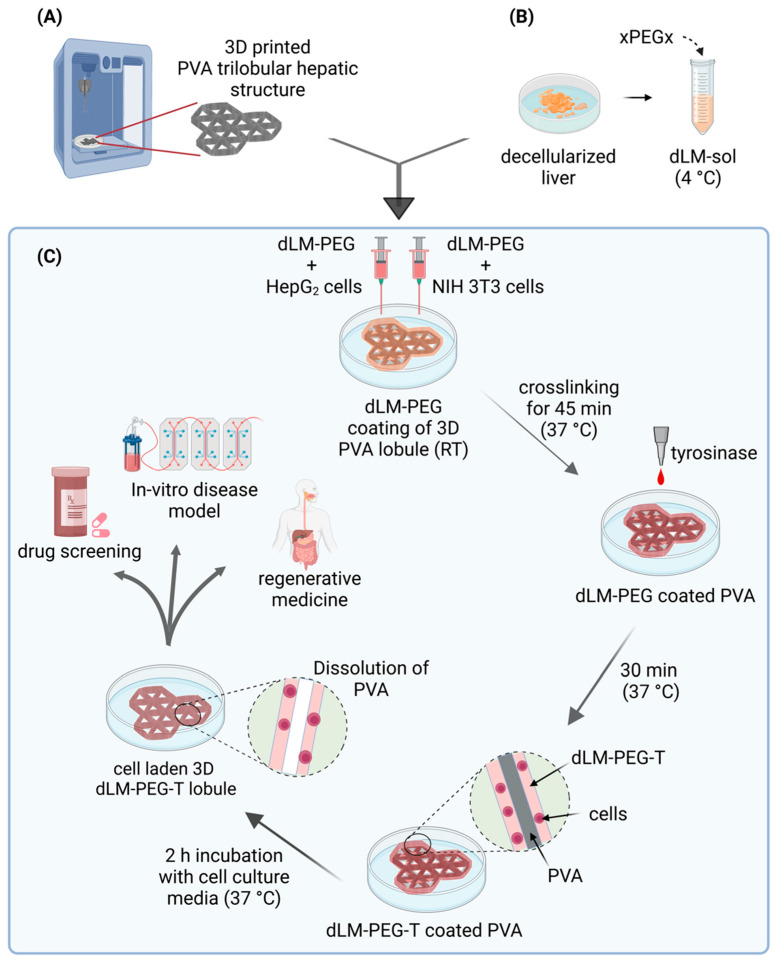
Schematic showing the development of dLM-PEG-T trilobular hepatic construct with indirect 3D printing of PVA. (**A**) PVA is 3D-printed into a trilobular hepatic structure, and (**B**) the dLM-sol is formed from decellularized liver tissue followed by the addition of polyethylene glycol (xPEGx, x = succinimidyl valerate). (**C**) The dLM-PEG formulations mixed with HepG_2_ and NIH 3T3 cells were dispersed on PVA and crosslinked at 37 °C, followed by the addition of tyrosinase to further enhance the crosslinking. The final dLM-PEG-T construct was incubated for 2 h to dissolve the PVA, leaving behind a 3D structure with lumen patency for applications in drug screening, in vitro disease models, and regenerative medicine.

In this study, we successfully developed a highly robust dLM-PEG-T (dLM crosslinked with xPEGx and mushroom tyrosinase) based trilobular hepatic scaffold with a 3D printed PVA sacrificial framework, as shown in Figure 1. The liver cancer cell line, HepG_2_, and fibroblast cell line, NIH 3T3, were embedded in dLM-PEG hydrogels separately and dispersed layer by layer over the 3D-printed PVA trilobular construct to crosslink at 37 °C with further addition of tyrosinase to enhance the mechanical properties and rheology. The PVA was dissolved and drained to transfer its 3D-printed architecture to the crosslinked dLM-PEG-T, leaving behind microchannels of 0.39 mm width. We investigated the PVA dissolution kinetics and the diffusion rate through the different thicknesses of dLM-PEG-T. We characterized the dLM-PEG-T before and after crosslinking with xPEGx and tyrosinase to observe its rheological behavior at 4 °C and 37 °C. The liver cell line HepG_2_ and the fibroblast cell line NIH 3T3 cells were used to study the biocompatibility for seven days. The trilobular model was fabricated with the layer-by-layer assembly of HepG_2_-embedded dLM-PEG-T and NIH 3T3-embedded dLM-PEG-T over 3D-printed PVA. The liver-specific protein markers were quantified with albumin secretion, urea secretion, and gene expression over 7 days. Here, we have attempted to replicate a scaled-up trilobular hepatic structure with lumen patency for media exchange.

## 2. Materials and Methods

### 2.1. Materials

PVA filament was purchased from 3DVerkstan (Ultimaker PVA, 2.85 mm, Solna, Sweden). The porcine liver was purchased from a slaughterhouse locally. Succinimidyl valerate-PEG-succinimidyl valerate (MW 5000) was purchased from Laysan bio-Inc, Arab, AL, USA, and mushroom tyrosinase (25 KU, ≥1000 unit per mg solid) was bought from Sigma-Aldrich AB (St. Louis, MO, USA). Unless specifically mentioned, all other chemicals used in this study were purchased from Sigma-Aldrich Sweden AB. All the cell culture reagents were procured from Thermo Fisher Scientific (Waltham, MA, USA). More details about other reagents are mentioned below.

### 2.2. 3D Printing of PVA

PVA was printed using Ultimaker 2 with a 0.6 mm and 0.4 mm nozzle to print a thin filament and a 3D trilobular hepatic construct at 180 °C. These 3D structures were printed in a sterile biological cabinet and sterilized under UV for 15–30 min. The PVA structure was stored at room temperature (RT) in a sealed sterile container with desiccant to avoid moisture. All the experiments involving PVA printing, handling of the structure, and cell culture were performed in a BSL-2 lab.

### 2.3. Preparation of dLM-PEG-T Hydrogel

According to our previous work [43], the porcine liver was decellularized and lyophilized to develop a 3–3.2% dLM solution with acetic acid and pepsin. This solution was adjusted to a pH value of 7–7.4 at a temperature around 4 °C and designated dLM-sol. The dLM-sol can be crosslinked at 37 °C into dLM-gel, but instead, xPEGx was introduced in dLM-sol as a crosslinking agent at a concentration of 50 mg/mL and mixed thoroughly while maintaining the low temperature. This formulation was crosslinked at 37 °C for 45 min and designated dLM-PEG. Once crosslinked, mushroom tyrosinase was added at a concentration of 800 units/mL of the dLM-PEG and further crosslinked for 30–40 min at 37 °C. All these formulations were characterized to understand their rheological behavior and crosslinking.

### 2.4. Rheology and Degree of Crosslinking

Rheological characterization was performed to understand the flow behavior of the different formulations at different temperatures. TA Instruments (Discovery Hybrid, New Castle, DE, USA ) rheometer with a 25 mm parallel plate setting was used at varying temperatures to evaluate the rheological properties of the formulations. Firstly, the samples were tested for their viscosity at 4 °C and 37 °C with a steady shear rate from 0.1 to 1000 s^−1^. The following formulations were tested at their respective temperatures: dLM-sol (4 °C), dLM-PEG (4 °C), dLM-gel (37 °C), dLM-gel (37 °C), and dLM-PEG-T (37 °C). Next, the dynamic frequency sweep was analyzed between 0.1–100 Hz to understand the change in storage and loss modulus. Except for dLM-sol, all the samples were analyzed for frequency sweep at a constant strain of 1% to observe frequency-dependent changes. Lastly, gelation kinetics of dLM-PEG and dLM-PEG-T was conducted at 37 °C to study the crosslinking time of the formulations for 2700 s.

A scanning electron microscopy (SEM) was used to study the morphology and internal microstructure of crosslinked formulations of dLM-gel, dLM-PEG, and dLM-PEG-T. The average pore size from the SEM images was calculated using ImageJ2 (Version: 2.3.0). Briefly, the hydrogels were frozen at −80 °C, lyophilized overnight, and sectioned into thin layers, followed by imaging in Hitachi TM-1000 (scanning electron microscope, Hitachi, Tokyo, Japan).

To analyze the degree of crosslinking, the total content of free amines present in non-crosslinked dLM-PEG (4 °C) was compared with crosslinked formulations of dLM-gel, dLM-PEG, and dLM-PEG-T using a Tri-nitro benzene sulfonic acid (TNBS) assay with a previously used protocol [43,44]. To 1.8 mg of sample, 4% NaHCO3 and 0.05% TNBS (5% *w*/*v*, Sigma-Aldrich AB, Sweden) were added at 40 °C. After 2 h, samples were hydrolyzed with 6 M HCl for 90 min at 60 °C, followed by measuring the absorbance at 320 nm.

### 2.5. Cell Culture and Encapsulation in dLM-PEG Scaffold

Human HepG_2_ (Sigma-Aldrich AB, Sweden) and mouse NIH 3T3 (DSMZ, Braunschweig, Germany) cell lines were maintained and expanded separately in complete media containing Eagle’s minimal essential medium (Gibco MEM, 110995080, Waltham, MA, USA), penicillin-streptomycin (Gibco, 15140122) and 10% fetal bovine serum (Gibco, A3160502) at a temperature of 37 °C and 5% CO_2_. Both the cell lines were passaged after every 3 days at about 90% confluency and dissociated from cell culture plates according to our previously used protocol [43]. After that, the cells were collected and resuspended in FBS (10%) separately. To prevent cells from osmotic shock inside dLM-PEG, 10x MEM was added at a concentration of 1/10th volume of the dLM-PEG. In the end, HepG_2_ cells and NIH 3T3 cells in 10% FBS were encapsulated at a density of 7–8 × 10^6^ cells/mL and 3–4 × 10^6^ cells/mL, respectively, in two separate dLM-PEG formulations containing 10× MEM and mixed gently at 4 °C temperature. Two dLM-PEG formulations containing HepG_2_ and NIH 3T3 cells separately were ready for use with the PVA construct.

To permanently label HepG_2_ and NIH 3T3 cells for tracing after generations, CellTrace CFSE (C34554, Invitrogen, Waltham, Massachusetts, USA) and CellTrace Violet (C34557, Invitrogen) fluorescent stains were used, respectively, according to the manufacturer’s protocol. Briefly, both the cell lines were incubated with their respective dyes at 37 °C and 5% CO_2_ for 15–20 min, followed by washing with PBS. The HepG_2_ and NIH 3T3 cells were resuspended separately in 10% FBS, followed by gently mixing the cells in two dLM-PEG formulations containing 10x MEM at 4 °C.

Over time, the changes in the co-culture system of HepG_2_ and NIH 3T3 cells embedded in dLM-PEG-T were compared to a monoculture system containing only HepG_2_ cells at a density of 7–8 × 10^6^ cells/mL of dLM-PEG-T. Collagen (type 1 rat tail, Sigma-Aldrich AB) was also used as a control to observe the co-culture system.

### 2.6. Preparation of Cell-Laden dLM-PEG-T Trilobular 3D Construct

The development of the PVA trilobular hepatic construct was initiated with the development of a non-complex dLM-PEG-T channel. A 3D-printed 0.6 mm PVA filament was coated with dLM-PEG-T and crosslinked. The coated filament was placed in PBS to dissolve the PVA and observed for its shape and form. The same filament was also used to create a micro-channel embedded in the crosslinked dLM-PEG pool in a Poly(methyl methacrylate) (PMMA) chip, as shown in Supplementary Materials, Figure S1A,B. A 3D mesh structure of PVA was also printed to observe its behavior and the internal architecture created with dLM-PEG (Supplementary Materials, Figure S2). Next, a more complex 3D structure with a trilobular hepatic design was 3D-printed with a 0.4 mm nozzle using Ultimaker 2 printer at 180 °C. The 3D-printed PVA trilobular hepatic construct was placed over a thin layer of acellular dLM-PEG-T spread in a well plate to attach the PVA base. For co-culture experiments, NIH 3T3 cell-laden dLM-PEG was dispersed with a sterile syringe of 400-micron diameter on top of the PVA structure’s strands and carefully avoided covering the triangular lumen for patency at RT for rapid crosslinking. Once the PVA structure was coated entirely, HepG_2_ cell-laden dLM-PEG was dispersed from a separate 400-micron diameter syringe over the NIH 3T3 cell-laden PVA construct as a second layer, and the structure was placed immediately inside the cell culture incubator at 37 °C and 5% CO_2_ for crosslinking. After 45 min, tyrosinase solution was added on top of the 3D construct and crosslinked for 30 min, followed by the addition of 2–3 mL of 1× complete MEM cell culture medium prepared as previously mentioned and placed back inside the cell culture incubator. The cell culture medium was replaced twice at 1 h, and a fresh medium was introduced again. To dissolve the PVA faster, the cell culture plate was swirled gently twice. Microscopic images were taken at different stages of the dLM-PEG-T structural development with PVA using a Dino-Lite digital microscope (New Taipei City, Taiwan), and the dimensions were analyzed. A similar protocol was followed for Celltrace labeled cells in two different dLM-PEG formulations to create a 3D trilobular hepatic construct.

Collagen (type I) was used as a control with a co-culture of HepG_2_ and NIH 3T3 cells at the same cell density as the dLM-PEG-T co-culture experiments. The co-culture, monoculture, and control groups were observed for the next 7 days for biocompatibility with the PVA, cell viability, and expression of liver-specific functions.

In parallel, an acellular dLM-PEG-T 3D trilobular construct was also developed to observe the final dimension of the construct and study its long-term stability through weight loss measurements at different time points. A microscopic image of the degraded structure was taken on day 21. We also examined the refractive index values of the dissolved PVA in PBS with time using a Rudolph refractometer (J157, Hackettstown, NJ, USA). After placing the dLM-PEG-T coated PVA structure in PBS, the supernatant (containing dissolved PVA) was collected at an interval of 30 min for the next 6 h and then at 24 h. The data were presented as a percentage of the difference between the refractive index of samples and PBS (control, no PVA). Along with this, we also examined the permeability of the dLM-PEG-T hydrogel by measuring the rate of diffusion of FITC-Dextran (MW 4000, Sigma-Aldrich, Saint Louis, MO, USA) over a layer (100, 400, and 600 μm) of dLM-PEG-T within a Transwell system (0.4 μm pore size, Corning, NY, USA), using a previously published protocol [45]. The migrated FITC-Dextran through the dLM-PEG-T layers was measured by detecting the fluorescence from the apical compartment in the Transwell after 1 h and 2 h at 490 nm excitation and 520 nm emission.

### 2.7. Cell Viability, Proliferation, and Metabolic Activity

Fluorescent staining of the dLM-PEG-T construct on days 1, 3, and 7 was performed to investigate their cell viability and proliferation using Calcein AM (2 μM/mL) for live cells and propidium iodide (4 μM/mL) for dead cells by washing with PBS and staining for 30 min. Celltrace-labeled HepG_2_ and NIH 3T3 cells embedded in dLM-PEG-T were also observed on day 3 by washing the constructs twice in PBS. A confocal microscope (Zeiss LSM900-Airy2) was used to obtain the images at 5×, 10×, and 20× resolutions. A tile scan was used to acquire a part of the dLM-PEG-T lobule on the x–y axis for live/dead assay at different time points. The entire dLM-PEG-T construct was also imaged on day 7 using the tile scan followed by stitching. Live and dead cells were counted in Imaris software 9.9.0 (Oxford Instruments, Abingdon, UK). The metabolic activity of the live cells was quantitatively measured using the alamarBlue^TM^ assay, in which the scaffolds were washed with PBS and treated with alamarBlue^TM^ for 3 h at 37 °C. The supernatant was collected, and fluorescence intensity was measured at an excitation of 540 nm and emission of 590 nm against a blank control with no cells. At the same time, a known number of cells with increasing concentration were treated with alamarBlue^TM^. A standard curve correlating the number of viable cells with the relative fluorescent units was obtained.

The migration of cells through a thick layer of dLM-PEG-T was microscopically analyzed at different time points for 24 h. The HepG_2_ and NIH 3T3 cells in serum-free MEM media were seeded on the dLM-PEG-T layer on the apical side of a Transwell, whereas the basal side was filled with 1× complete MEM media (as mentioned above) to direct the cell migration or invasion through the hydrogel.

### 2.8. Functional Assessment and Gene Expression Analysis

Cell-culture media from dLM-PEG-T constructs and control were collected at different time points. The albumin and urea levels were quantified using the Human Albumin ELISA kit (ab179887, Abcam, Cambridge, UK) and Urea Assay Kit (ab83362, Abcam), according to the manufacturer’s protocol. The number of viable cells (only HepG_2_) at a particular time point was correlated to the expression of albumin and urea. Initially, the ratio of the embedded HepG_2_ and NIH 3T3 cells was 2:1. Thus, it was assumed that this ratio would be the same at each time point during the experimental measurements.

For evaluating the mRNA gene expression, RNA was extracted from dLM-PEG-T constructs and the control at different time points using the RNeasy Mini kit from Qiagen (Hilden, Germany). The cDNA was synthesized using the High-Capacity cDNA Reverse Transcription Kit (4368814) protocol. RT-qPCR was performed with a StepOnePlus PCR instrument and SYBR™ Green PCR Master Mix (4309155). All the results were normalized to the expression of the housekeeping gene glyceraldehyde-3-phosphate dehydrogenase (GAPDH) and compared to the mRNA expression levels of the target genes.

### 2.9. Statistical Analysis

All of the statistical analysis was performed in GraphPad Prism (version 9) using two-way ANOVA with Bonferroni correction. All measurements were performed in triplicates and expressed as the standard error of the mean. The difference was considered statistically significant with *p* value < 0.05, 0.01, 0.001, and 0.0001 denoted by *, **, *** and ****, respectively.

## 3. Results and Discussion

### 3.1. Preparation and Characterization of dLM-PEG-T Hydrogel

**Figure 2:**
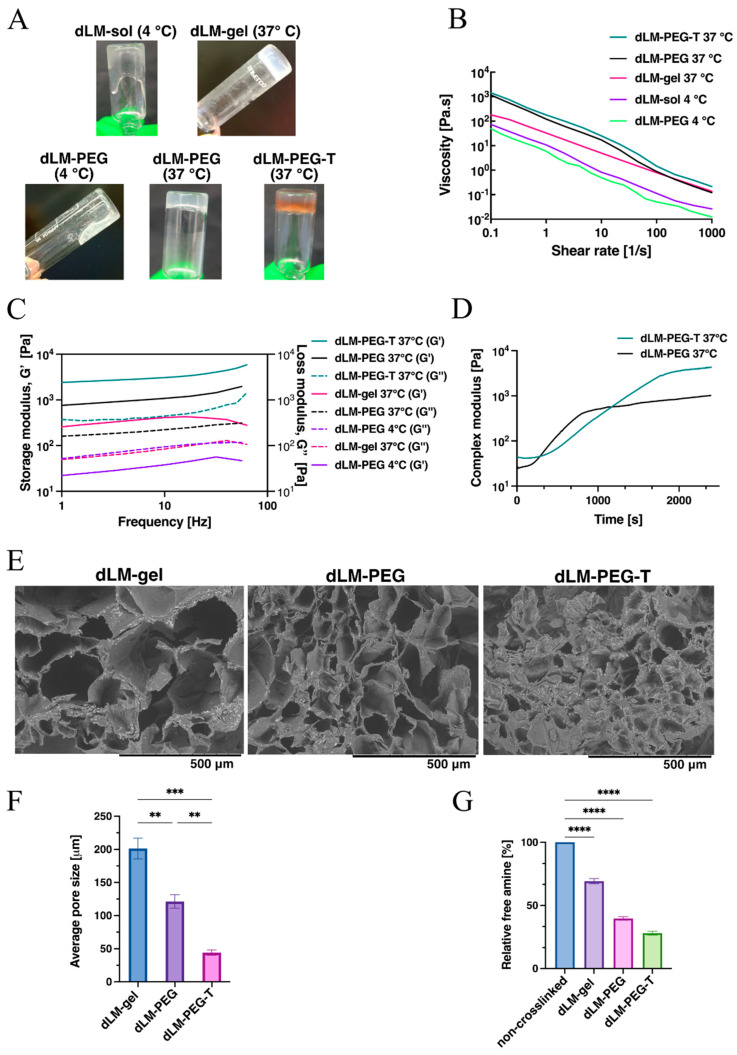
Investigation of dLM formulations (**A**) visually with tube inversion behavior of dLM-sol (4 °C), dLM-gel (37 °C), dLM-PEG (4 °C), dLM-PEG (37 °C), and dLM-PEG-T (37 °C) followed by (**B**) their viscosity analysis with increasing shear rate. (**C**) Oscillatory frequency sweep at 1% strain to compare storage and loss modulus and (**D**) gelation kinetics of dLM-PEG and dLM-PEG-T at 37 °C. (**E**) SEM images of the cross-section of crosslinked formulations of dLM-gel, dLM-PEG and dLM-PEG-T (scale bar 500 μm) and their (**F**) average pore size (*n* = 3, ** *p* < 0.01 and *** *p* < 0.001). (**G**) TNBS assay with relative free amine percentage across all crosslinked formulations relative to non-crosslinked formulations (*n* = 3, **** *p* < 0.0001). Error bars represent the standard error of the mean.

The decellularization of the porcine liver tissue was carried out according to our previously published work [43]. After digestion with pepsin and acetic acid into a 3–3.2% concentration, dLM is in the form of free-flowing hydrogel with a low pH value of 3. Thus, the pH is maintained at around 7.0–7.4 at 4 °C and designated dLM-sol. Once the pH is adjusted to physiological conditions, the dLM hydrogel becomes temperature-sensitive, crosslinking rapidly at 37 °C, and this hydrogel is designated as dLM-gel. However, in our study, a high concentration of xPEGx was introduced in dLM-sol at 4 °C to enhance the rheological properties, and it was further crosslinked at 37 °C for 45 min and designated as dLM-PEG. For instant gelation and denser crosslinking, 800 units/mL of tyrosinase was introduced as a secondary crosslinker in dLM-PEG to further improve the robustness, and it was designated as dLM-PEG-T. The tube inversion behavior of the hydrogel formulations was used to observe their robustness and test their suitability for further evaluation (Figure 2A). A higher concentration of xPEGx and tyrosinase was used in this study compared to our previous work, as more crosslinking was required to improve the robustness of dLM hydrogel without introducing any rheology enhancer such as gelatin [43]. It was observed that all the non-crosslinked formulations at lower temperatures (dLM-sol and dLM-PEG) behaved as soft spreadable gels, but once they were incubated at 37 °C, they behaved as a crosslinked gel. Visually, all the formulations show enhanced robustness after crosslinking, as they were not free-flowing in the tube. Tyrosinase additionally gave a light brownish color because of its enzymatic activity.

All the formulations mentioned above were quantified for their rheological behavior with viscosity measurements at different temperatures. Decreasing viscosity with increasing shear rate across all the formulations was observed, demonstrating shear thinning behavior (Figure 2B). Thus, all the formulations were non-Newtonian and, therefore, cell-friendly due to low viscosity at a high shear rate. Non-crosslinked formulations of dLM-PEG and dLM-sol at 4 °C had the lowest viscosity. In contrast, the crosslinked formulations at 37 °C had higher viscosity, implying that crosslinking enhances the thickness of all the formulations. At 1 s^−1^ shear rate, the viscosity of dLM-PEG-T is 182.7 ± 1.6 Pa·s, which is 15.5 times higher than the viscosity of dLM-sol (11.8 ± 1.56 Pa·s) and 30 times higher than dLM-PEG (6 ± 0.45 Pa·s) at 4 °C (Supplementary Materials, Figure S3A). However, after crosslinking with xPEGx, the viscosity of dLM-PEG-T was 1.7 times higher than dLM-PEG at 37 °C, implying a substantial improvement in viscosity with xPEGx addition in dLM-sol. Since dLM-sol and dLM-PEG exhibited similar viscosity and flow behavior at low temperatures, only dLM-PEG was analyzed further for other rheological properties. This study allowed us to determine the best formulation for encapsulating cells (dLM-PEG) with the lowest viscosity and easy extrudability. On the other hand, dLM-PEG-T was the superior formulation with the highest viscosity (dLM-PEG-T) and the potential to support a 3D structure.

Further evaluation of the storage (G’) and loss (G’’) modulus revealed the liquid-like behavior of dLM-PEG at 4 °C with a higher loss modulus than the storage modulus (Figure 2C). However, when all the formulations were incubated at 37 °C, the rheological properties improved with crosslinked hydrogels, displaying a higher elastic effect of storage modulus than the loss modulus. The formulations can retain their form and shape with higher storage modulus, which is the prerequisite for multi-scaling a structure. All the formulations were stable throughout the tested frequency range with a mild increment. Like the results obtained from viscosity measurements, the dLM-PEG-T formulation illustrated the highest storage modulus of 2554 ± 82.1 Pa at 1 Hz, which is 120 times higher than non-crosslinked dLM-PEG at 4 °C and 3.3 times higher than crosslinked dLM-PEG at 37 °C (Supplementary Materials, Figure S3B). Thus, dLM-PEG, after crosslinking, is a robust hydrogel with high viscosity and modulus. Still, the addition of tyrosinase further strengthens the rheological properties for better shape fidelity of the 3D structure. The gelation kinetics of dLM-PEG and dLM-PEG-T was also evaluated at 37 °C showing an increase in complex modulus over time and indicating gelation (Figure 2D). After 30 min and 40 min, the modulus of dLM-PEG and dLM-PEG-T reached a plateau with time, indicating complete crosslinking. Due to the presence of tyrosinase, dLM-PEG-T can be concluded as a stiffer hydrogel with increased mechanical properties for long-term stability. Thus, tyrosinase enhanced the improvements in rheological properties imparted by adding xPEGx in dLM-sol.

The cross-sectional microstructures of all the crosslinked formulations were studied with SEM (Figure 2E) to observe changes in pore size and morphology with increasing crosslinking. All the micrographs showed varying pore sizes with interconnectivity and a similar orientation. The pore size decreased with the introduction of xPEGx, which was further reduced with the addition of tyrosinase. The average pore sizes were 211 ± 5.9 µm for dLM-gel, 115 ± 5.2 µm for dLM-PEG and 44 ± 4 µm for dLM-PEG-T (Figure 2F). The dLM-PEG-T displayed tiny pores with complex tighter microarchitecture, implying high crosslinking compared to weakly crosslinked dLM-gel with huge pores. The similarity in rheological investigation and SEM results was also confirmed with the TNBS assay. The crosslink density of dLM formulations was evaluated by quantifying the decreasing concentration of free amines resulting from the crosslinking reactions (Figure 2G). All the results were relative to the non-crosslinked dLM-PEG at 4 °C and assumed to contain 100% of the available free amine groups. The lowest concentration of free amines was demonstrated by dLM-PEG-T with only 28 ± 1.51% free amines, followed by dLM-PEG with 39.7 ± 1.4%. dLM-gel showed 69.2 ± 2% free amines, implying a comparatively low degree of crosslinking. Finally, the rheology, SEM, and TNBS assay results were consistent in validating dLM-PEG-T with the highest viscosity, storage modulus, and high degree of crosslinking for further analysis to develop the 3D trilobular hepatic construct. On the other hand, dLM-PEG at 4 °C exhibited the lowest rheological properties and a low degree of crosslinking. Previous work has shown that biomaterials with a lower storage modulus and concentration are more cell-friendly and improve cell viability and proliferation, thus validating dLM-G at 4 °C for cell encapsulation before the addition of tyrosinase.

### 3.2. Development of 3D Trilobular Hepatic Construct with PVA and dLM-PEG-T

**Figure 3:**
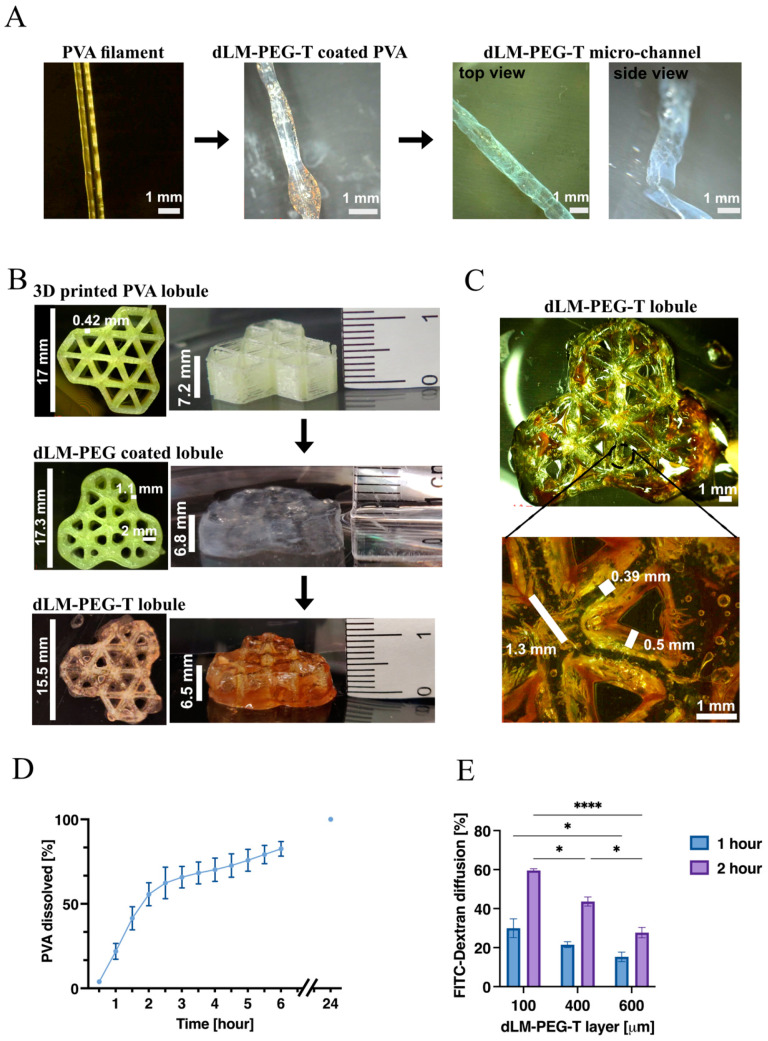
Development of the 3D trilobular construct with dLM-PEG-T, initiated with (**A**) the formation of a microchannel by coating the 3D-printed PVA filament. The PVA dissolved, forming a hollow tubular structure with dLM-PEG-T (scale bar 1 mm). (**B**) 3D printing of a PVA trilobular structure followed by coating it with dLM-PEG at RT and further addition of tyrosinase to form a crosslinked dLM-PEG-T trilobular construct with their respective dimensions. (**C**) Representative cross-sectional image of the dLM-PEG-T trilobular structure (top view) and its microscopic image with the line width and spacing (scale bar 1 mm). (**D**) PVA solubility kinetics (*n* = 3) and (**E**) quantification of FITC-Dextran diffusion in 1 h and 2 h through the increasing thickness of dLM-PEG-T compared to the diffusion through an empty Transwell (*n* = 3, * *p* < 0.05 and **** *p* < 0.0001). Error bars represent the standard error of the mean.

The essential step in understanding the behavior of PVA with dLM-PEG-T hydrogel was initiated by developing a less intricate dLM-PEG-T hollow micro-channel (Figure 3A) by coating the 3D-printed PVA with dLM-PEG-T. The PVA dissolved, leaving behind a 3D dLM-PEG-T open tubular structure with a 0.7–0.8 mm diameter, which was lyophilized to observe the hollow shape (Supplementary Materials, Figure S4). This channel was subjected to various movements in aqueous media but maintained its shape and form (Supplementary Video S1). Next, a microfluidic chip with the PVA filament (0.6mm) embedded in a pool of crosslinked dLM-PEG was developed to create a micro-channel in a dLM environment (Supplementary Materials, Figure S1B). The PVA filament dissolved and formed a hollow micro-channel with a continuous flow of a medium from one inlet to the other outlet (Supplementary Video S2). This microfluidic chip was unclogged and robust, further validating the sturdiness of the channels formed with this technology (Supplementary Materials, Figure S1C).

Based on these results, we attempted to create a more complex 3D structure relevant to the liver model. We successfully developed a trilobular hepatic model with HepG_2_ and NIH 3T3 cells embedded in the cell-friendly dLM-PEG at RT, coating the 3D-printed PVA. The PVA structure was 17 mm in length and 7.2 mm in height with a line width of 0.42 mm (Figure 3B) with triangular-shaped gaps showing lumen patency. After dispersing the HepG_2_ and NIH 3T3 cells embedded dLM-PEG formulations over PVA, the dimensions changed to 17.3 mm length, 1.1 mm line width, and 2 mm spacing of the lumen patency (Figure 3B). The increase in the dimension was due to the additional layers of dLM-PEG hydrogels over and around the PVA strands in the structure. The coated PVA trilobular structure was crosslinked immediately at 37 °C. Notably, the PVA in the structure started to dissolve due to the water content of the hydrogel and became fragile. Handling this 3D construct high on the z-axis was challenging due to the comparatively soft nature of the dLM-PEG. To avoid collapsing the 3D structure because of its weight, tyrosinase solution was introduced as a secondary crosslinker immediately to stiffen the gel into a self-standing robust dLM-PEG-T trilobular structure with 15.5 mm length and 6.5 mm height (Figure 3B). There is an overall dimensionality decrement in the size and height of the final dLM-PEG-T construct from dLM-PEG, which might be due to additional crosslinking with tyrosinase leading to a small amount of shrinkage (Figure 3C). The microscopic images of the dLM-PEG-T construct after the complete dissolution of PVA revealed the actual line width of around 0.5 mm formed with dLM-PEG-T and 0.39 mm width of the microchannels. The center of the dLM-PEG-T lobule resembles the shape of the central vein of a liver with a lumen patency of 1.3 mm (Figure 3C). Thus, the line width of the 3D-printed PVA lobule decreased after dissolution from 0.42 mm to 0.39 mm in the final structure. This 3D construct exhibited good shape fidelity without excessive spreading, despite the weight of the formulation. Notably, these dimensions of the construct are of substantial magnitude compared to the in vivo hepatic lobules. However, this is a unique attempt to develop a scaled-up model such as the liver to open avenues for future miniaturized studies. During the entire process, the 3D construct was incubated at 37 °C with 5% CO_2_ to maintain cell-friendly conditions within the dLM-PEG and dLM-PEG-T.

The dissolution kinetics of PVA was analyzed to understand the time-dependent behavior of dLM-PEG-T coated PVA (Figure 3D). The refractive index of samples (PVA containing supernatant) collected every 30 min exhibited a sharp increase in the refractive index for the first 3–4 h, implying rapid dissolution. More than 60% of the PVA was dissolved between 3–4 h, after which it stabilized, and a slight increase was observed for 6 h with almost 80% PVA dissolved. The readings obtained after 24 h and 48 h showed the same refractive index, suggesting complete dissolution of PVA between 6 and 24 h. Additionally, the diffusion through the different thicknesses of dLM-PEG-T was examined, as the composition must allow diffusion of oxygen, nutrients, and other proteins (Figure 3E). The permeability assay mimicked the absorption and exchange of solutes (mol wt 4000) from the cell culture media and showed an inversely proportional correlation with increasing thickness. The results were compared to the 100% control to measure the relative diffusion rates after 1 h and 2 h. As expected, 100 μm thickness revealed the highest diffusion rate increasing from 29.9± 4.84% to 60 ± 0.0.86%, followed by 400 μm thickness, increasing from 21.5 ± 1.51% to 43.6 ± 2.33% in a period of 1 h and 2 h, respectively. As expected, the lowest diffusion rate was displayed by the 600 μm thickness with 15 ± 0.2.43% in 1 h and 27.7 ± 2.68% in 2 h, revealing thickness-restrictive diffusion in dLM-PEG-T.

In this study, we dispersed the dLM-PEG non-crosslinked hydrogel over the strands of the PVA structure to obtain lumen patency in the final construct. However, we also tried to disperse the dLM-PEG hydrogel all over the PVA structure to submerge it in dLM-PEG-T (Supplementary Materials, Figure S5). But this study did not provide any information about the long-term changes in the structure obstructing the view of internal architecture (Supplementary Materials, Figure S6), with only micro-channels visible throughout. Thus, it was not explored for further applications. On the other hand, the dLM-PEG-T dispersed around the PVA strands exhibited good stability and robustness for 7 days, as shown in Supplementary Materials, Figure S7A, without any microscopic changes. However, on day 21, the structure was slightly deformed upon movements in the well plates and became very sensitive toward motion in the cell-culture media. The weight loss measurements at different time points displayed a reduction of almost 40% of the weight of dLM-PEG-T by day 21 (Supplementary Materials, Figure S7B). Microscopic images on day 21 revealed fragile lobules becoming segregated from the trilobular structure. Some previous work has shown similar results of fast degradation of the dLM [7,43]. Thus, a 7-day-long characterization of dLM-PEG-T was performed by embedding HepG_2_ and NIH 3T3 cells to examine the biocompatibility and liver-specific functions.

### 3.3. Cell Viability and Proliferation within the dLM-PEG-T Construct

**Figure 4:**
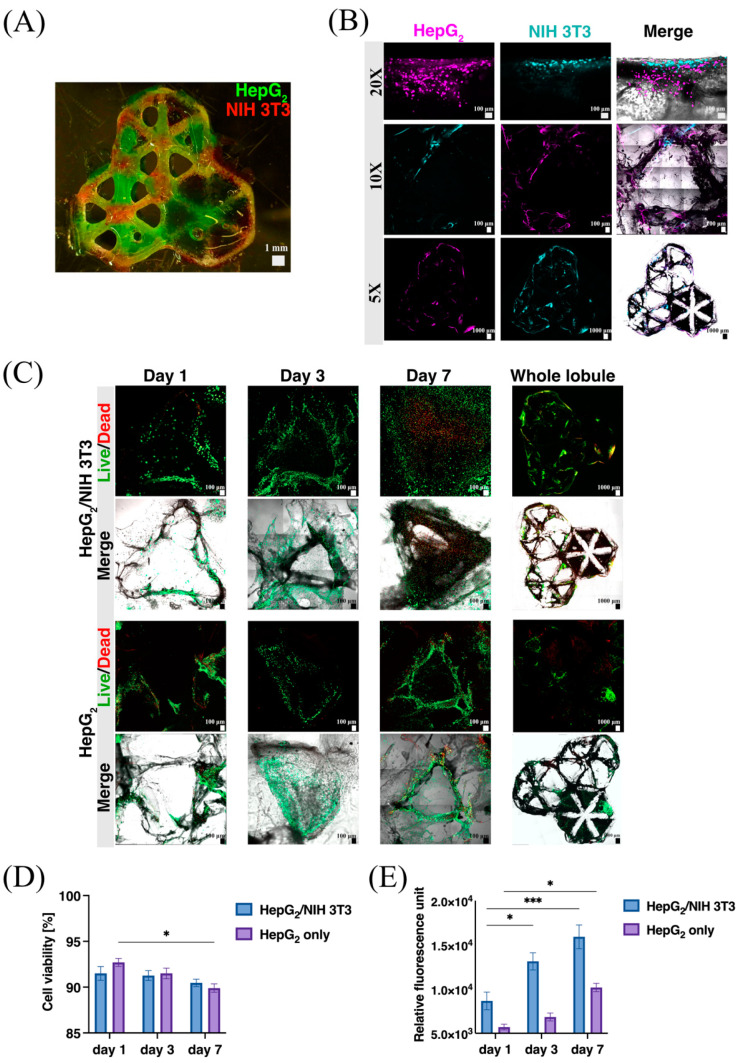
Cell biocompatibility and viability study. (**A**) Representation of co-culture of HepG_2_ (green) and NIH 3T3 (red) cells in the dLM-PEG-T trilobular structure. (**B**) Microscopic images of CellTracker labeled HepG_2_ and NIH 3T3 cells embedded in dLM-PEG-T with 20× and 10× magnification (scale bar 100 μm) together with the whole lobule (scale bar 1000 μm) at day 3 represented in different fluorescence channels. (**C**) Live/dead assay microscopic images on days 1, 3, and 7 (scale bar 100 μm) with the whole trilobular structure on day 7 (scale bar 1000 μm) compared to the monoculture of HepG_2_ cells. Comparison of co-culture of HepG_2_/NIH 3T3 with only HepG_2_ cells by (**D**) cell viability (*n* = 3, * *p* < 0.05) and (**E**) alamarBlue^TM^ assay at days 1, 3 and 7 (*n* = 3, * *p* < 0.05 and *** *p* < 0.001). Error bars represent the standard error of the mean.

To examine the functionality and biocompatibility of the 3D dLM-PEG-T hepatic construct created with PVA, the co-culture and monoculture of HepG_2_ cells were observed for their viability and proliferation for a period of 7 days. Previous studies have shown the importance of a co-culture environment for the superior viability and functions of liver cells [46,47] over monoculture. For co-culture studies, the contact between HepG_2_ embedded dLM-PEG-T and NIH 3T3 cells embedded in dLM-PEG-T was maximized by completely dispersing one of the lobules in the trilobular hepatic structure with subsequent layers of the co-cultured cells embedded in dLM-PEG-T formulations (Figure 4A). The increased cell-to-cell contact between HepG_2_ and NIH 3T3 cells were analyzed to understand the effect of co-culture interactions on the behavior of HepG_2_ cells. The HepG_2_ and NIH 3T3 cells were labeled with CellTracker to visualize the proliferation of each cell line and imaged on day 3. More HepG_2_ cells were visible, with higher distribution across the dLM-PEG-T surrounding the NIH 3T3 cells (Figure 4B). The trilobular structure demonstrated the distribution of each cell line separately and maintained its overall shape. Further, the live/dead assays performed on days 1, 3, and 7 are illustrated in Figure 4C for both HepG_2_/NIH 3T3 co-culture and HepG_2_ monoculture, showing viable cells with increasing cell populations with time. On day 7, the HepG_2_/NIH 3T3 co-culture was highly spread compared to the HepG_2_ cells. The images of the whole trilobular construct were captured on day 7 to show the integrity and cell distribution towards the end of our study.

The cell viability was high on day 1 for both HepG_2_/NIH 3T3 (91.5%) and only HepG_2_ (92.7%) cells, but it dropped slightly by day 7 with 90.4% for HepG_2_/NIH 3T3 co-culture and 89.9% for the monoculture (Figure 4D). Thus, no significant difference between the cell viability in co-culture and HepG_2_ cells was observed, as they both maintained a high number of live cells for 7 days. However, the alamarBlue^TM^ assay showed a higher metabolic activity for HepG_2_/NIH 3T3 cells with a steady rise in fluorescence with a 1.8-fold increase from day 1 to day 7 because of higher cell numbers in the co-culture system compared to the monoculture system (Figure 4E). The fluorescence intensity measured was almost double for HepG_2_/NIH 3T3 at each time point compared to the HepG_2_ cells. An apparent increase in metabolic functions was also visible in HepG_2_ monoculture, with a 1.7-fold increase from day 1 to day 7. This indicates that the dLM-PEG-T structure formed with PVA is biocompatible in both conditions and provides a supportive microenvironment for cell growth and proliferation. Similar results were observed in the co-culture with collagen control (Supplementary Figure S8A,B). The migration of HepG_2_ and NIH 3T3 cells through the dLM-PEG-T barrier revealed that the cells could migrate into the hydrogel within 6 h of the cell seeding (Supplementary Figure S9). This can further improve the application of co-culture studies in the investigation of cancer metastasis.

### 3.4. Liver-Specific Functions and Gene Expression

**Figure 5:**
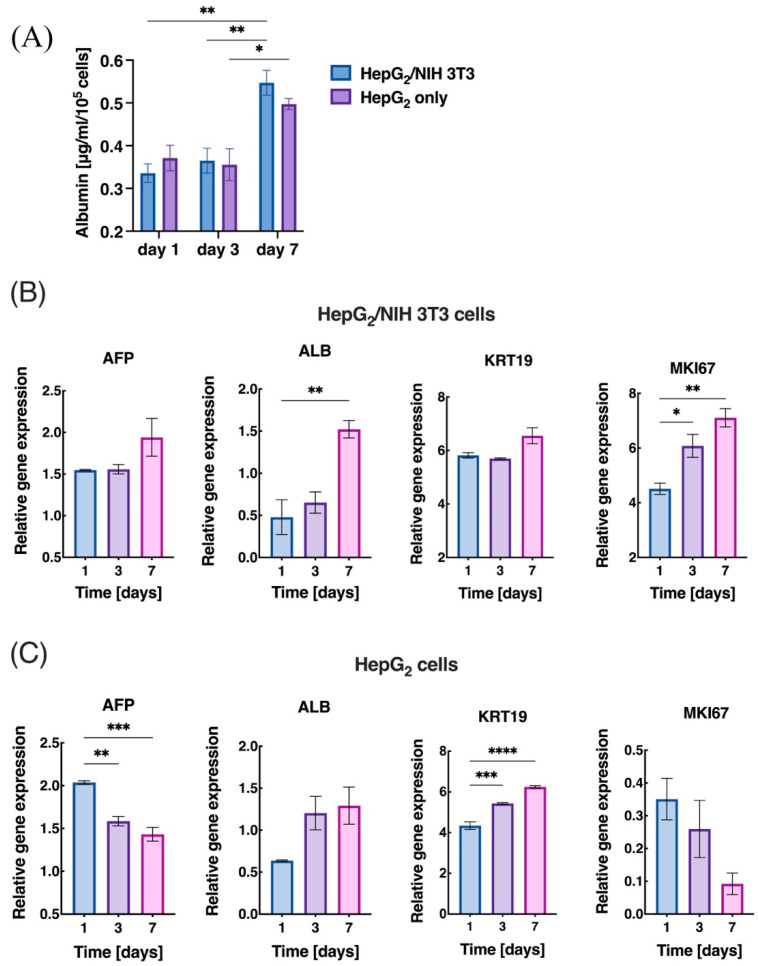
Expression of liver-specific functions in (**A**) co-culture of HepG_2_/NIH 3T3 cells compared to only HepG_2_ cells with Albumin secretion in μg/mL/10^5^ cells (*n* = 3, * *p* < 0.05 and ** *p* < 0.01) and gene expression analysis of (**B**) HepG_2_/NIH 3T3 cells (*n* = 3, * *p* < 0.05 and ** *p* < 0.01) compared to (**C**) HepG_2_ cells (*n* = 3, ** *p* < 0.01, *** *p* < 0.001 and **** *p* < 0.0001) at day 1, 3, and 7 for liver-specific genes AFP, ALB, KRT19, and MKI67. Error bars represent the standard error of the mean.

We investigated the effect of co-culture more extensively by characterizing liver-specific metabolic functions by analyzing the albumin production on days 1, 3, and 7 (Figure 5A). A 1.7-fold increase in the albumin expression from day 1 (0.335 ± 0.02 μg/mL) to day 7 (0.561 ± 0.03 µg/mL) in HepG_2_/NIH 3T3 cells was observed, whereas the HepG_2_ cells increased by 1.2-fold from day 1 (0.371 ± 0.03 µg/mL) to day 7 (0.487 ± 0.01 μg/mL). An increase in albumin expression with HepG_2_ cells is in line with our previously published work [48] and a characteristic observed in the co-culture of cells. Both the groups maintained a high level of albumin levels that drastically jumped from day 1 to day 7 because of the rapid proliferation of the cells. However, no significant increase in albumin concentration between days 1 and 3 was observed in the HepG_2_-only group. The collagen co-culture control maintained the albumin secretion throughout the 7 days of culture with an insignificant increase (Supplementary Materials, Figure S8C). Similarly, after culture for 7 days, the amount of urea was significantly higher in both groups, with a 4-fold increase from day 1 to day 7 (Supplementary Materials, Figure S10). The co-culture group maintained a higher and more sustained level of urea secretion than the monoculture, but both groups showed a sharp increase toward day 7. Hence, the co-culture in dLM-PEG-T showed a slightly higher albumin and urea expression towards day 7 than the monoculture group.

To further characterize the liver-specific phenotype of our cells system, we performed a thorough characterization of liver-specific genes and observed changes in the mRNA levels of hepatic markers AFP, ALB, MKI67, and KRT19 within the dLM-PEG-T construct for a period of 7 days. In HepG_2_/NIH 3T3, the expression of AFP, ALB, and MKI67 was increasing from days 1, 3, and 7; however, KRT19 displayed lower expression on day 3 with no significant change, but it was substantially increased on day 7 (Figure 5B). Thus, in the co-culture group, there was a significant increase in the expression of all the hepatic markers between day 1 and day 7. On the other hand, in HepG_2_-only cells, the expression of ALB and KRT19 showed an increase from days 1, 3, and 7, but AFP and MKI67 showed a constant decrease from day 1 onwards (Figure 5C). In the collagen control samples, the co-culture cells showed similar behavior with decreasing expression of AFP and MKI67 and a minimal increase in expression of ALB from day 3 to day 7. KRT19 mRNA expression appeared to be increased across all three samples irrespectively. The overall results exhibited improved expression of the four hepatic markers in HepG_2_/NIH 3T3 co-culture compared to the HepG_2_-only cells after 7 days. Collagen control also exhibited an increase in mRNA expression of all four hepatic markers from day 1 to day 7. However, the expression on day 3 was not significantly higher than on day 1 (Supplementary Materials, Figure S8D). This enhancement in the liver-specific metabolic activity and mRNA expression could be attributed to the co-culture system of HepG_2_ and NIH 3T3 cells, as observed in some previous studies as well [49,50] over the monoculture.

In this work, we attempted to develop a cytocompatible 3D system with PVA and dLM-PEG-T hydrogel. Additionally, we fabricated a liver-tissue model using a co-culture of HepG_2_ and NIH 3T3 cells and presented the vast potential of this indirect 3D-printed dLM-PEG-T model that permits the usage of more than one cell type. The collagen control for co-culture, however, formed a soft gel even after crosslinking, which was challenging to handle in 3D conditions. This implies that the crosslinking of ECM components is crucial for a sustainable 3D structure. For developing in vitro liver models in future studies, primary hepatocytes can be employed with multiple cell types such as Kupffer, Stellate, or endothelial cells to create a liver-like microenvironment in the dLM-PEG-T lobule with zonation and study their liver-specific functions [49,51,52]. The next phase of our research will focus on developing the intrinsic microchannels since vascularization is a prerequisite for the clinical translation of any tissue model [53]. It will include a functional liver lobule within a dLM-PEG microfluidic chip with sacrificial PVA (Supplementary Materials, Figure S11) to grow primary hepatocytes in a dynamic environment with a continuous flow of media and nutrients. 

## 4. Conclusions

In this work, indirect 3D printing of the PVA framework was used as sacrificial support to create a dLM-PEG-T trilobular hepatic construct with lumen patency for co-culture of HepG_2_ and NIH 3T3 cells with good cell viability and spreading. This is a unique strategy to create complex structures such as microchannels, microfluidic chip assembly or a 3D cell-culture system high in the z-axis with multiple cell types, as shown in this work, along with a range of biomaterials. Here, we established a robust 3D dLM-PEG-T hydrogel with high crosslinking and cytocompatible gelation with superior rheological properties and diffusion. By comparing the co-culture of HepG_2_/NIH 3T3 cells with a monoculture of HepG_2_ cells in dLM-PEG-T, we demonstrated a strong influence of fibroblasts on the liver cell line for a period of 7 days with increased albumin secretion and a consistent hepatic gene expression in vitro. We expect that these findings can be applied to generate a clinically relevant complex liver model with multiple cells based on zonation and dynamic conditions for drug screening and clinical exploration.

## Data Availability

All data are contained within the article.

## Supplementary Materials

The following supporting information can be downloaded at: https://www.mdpi.com/article/10.3390/bioengineering9110603/s1, Figure S1: Microfluidic chip with Poly(methyl methacrylate (PMMA) and dLM-PEG hydrogel pool with PVA embedded micro-channel, Figure S2: 3D-printed PVA grid structure with dLM-PEG, Figure S3: The viscosity and storage modulus of different formulations at low (1 Hz) frequency, Figure S4: Channel formation with dLM-PEG-T after PVA dissolution (side view), Figure S5: Trilobular structure with dLM-PEG filled inside, Figure S6: Side view of the trilobular structure with dLM-PEG filled inside on day 0, day 7, and day 21, Figure S7: Long term culture and degradation of dLM-PEG-T structure, Figure S8: The collagen control used as co-culture control with HepG2 and NIH 3T3 cells, Figure S9: Migration study of HepG_2_ and NIH 3T3 cells seeded over 600 µm thick dLM-PEG-T hydrogel at different time points, Figure S10: Urea secretion by the co-culture of HepG2 and NIH 3T3 cells, Figure S11: Liver lobule-on-chip concept for future studies; Supplementary Video S1: Micro-channel in PBS solution, Supplementary Video S2: Micro-channel in dLM chip with perfusion
