# Supplementary material for: Indirect 3D Bioprinting of a Robust Trilobular Hepatic Construct with Decellularized Liver Matrix Hydrogel
Source: Bioengineering (Basel). 2022 Oct 22;9(11):603. doi: 10.3390/bioengineering9110603 (PMC9687301; doi:10.3390/bioengineering9110603)
Supplement: Supplementary file 1 [file bioengineering-09-00603-s001.zip › bioengineering-1945263 supplementary for conversion v3.pdf]

bioengineering

Article

Indirect 3D Bioprinting of a Robust Trilobular Hepatic Construct with Decellularized Liver Matrix Hydrogel

Vamakshi Khati [1], Johannes Artturi Turkki [2], Harisha Ramachandraiah [3], Falguni Pati [4], Giulia Gaudenzi [1,5] and Aman Russom [1,6,*]

Figure S1. Microfluidic chip with Poly(methyl methacrylate (PMMA) and dLM-PEG hydrogel pool with PVA embedded micro-channel.

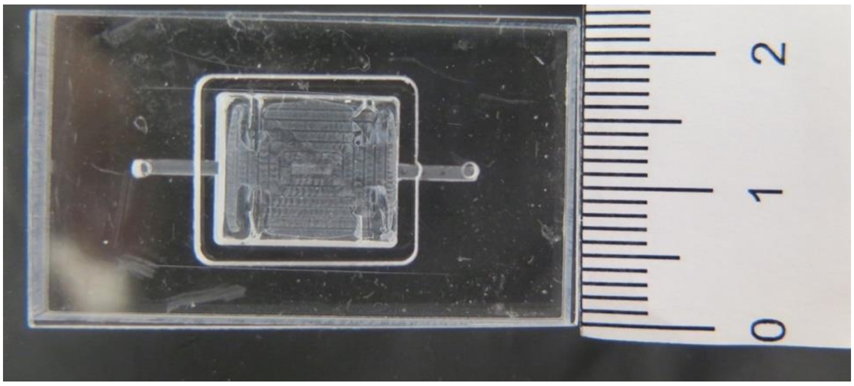

Figure S1. (A) PMMA microfluidic chip with 11 mm length, 13 mm width, and 2 mm height.

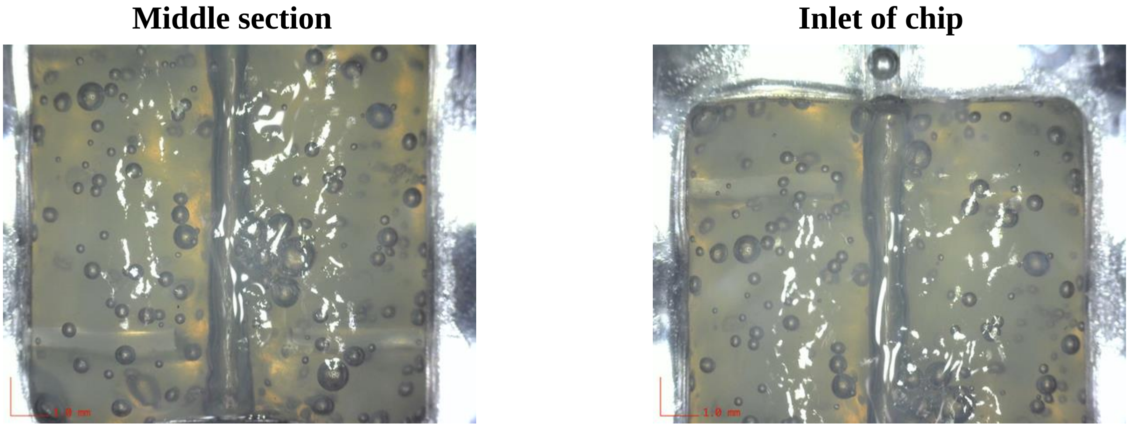

Figure S1. (B) PMMA microfluidic chip filled with dLM-PEG. The PVA filament is connected to the etched micro-channel in PMMA. Microscopic images of the dLM-PEG chip.

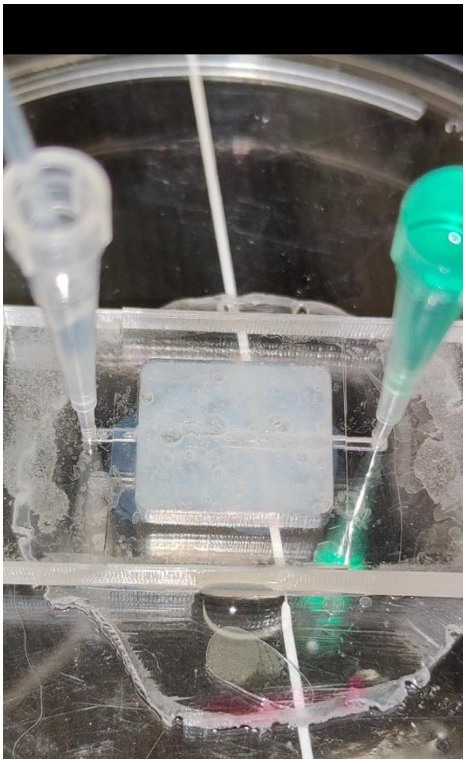

Figure S1. (C) PMMA microfluidic chip with dLM-PEG pool and a micro-channel created with dissolved PVA. The green solution was passed through the channel to show the robustness of the system.

Figure S2. 3D printed PVA grid structure with dLM-PEG.

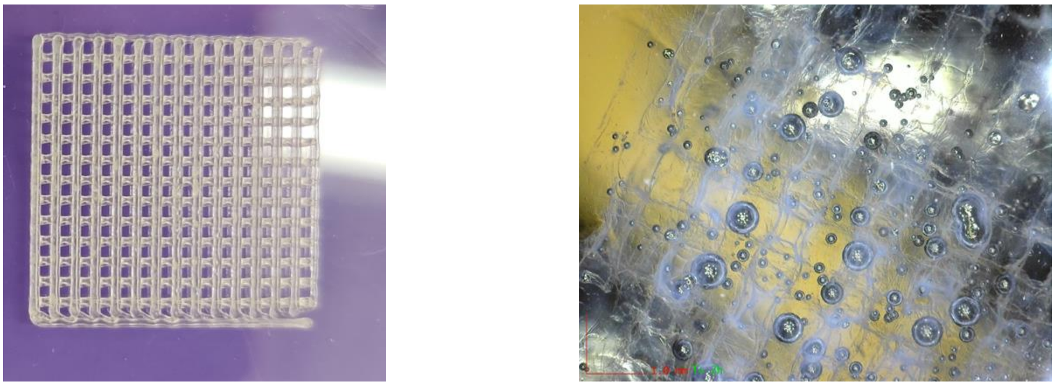

Figure S2. 3D printed PVA (left) and the dLM-PEG structure formed after the addition of dLM-PEG at 4 °C and crosslinking at 37 °C (right).

Figure S3. The viscosity and storage modulus of different formulations at low (1 Hz) frequency.

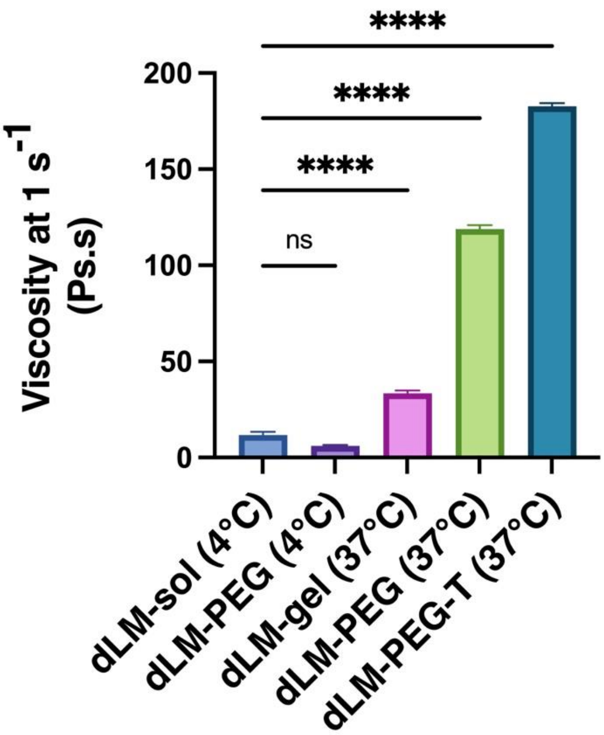

Figure S3. (A) The viscosity of different formulations at 1 s^{-1} shear rate.

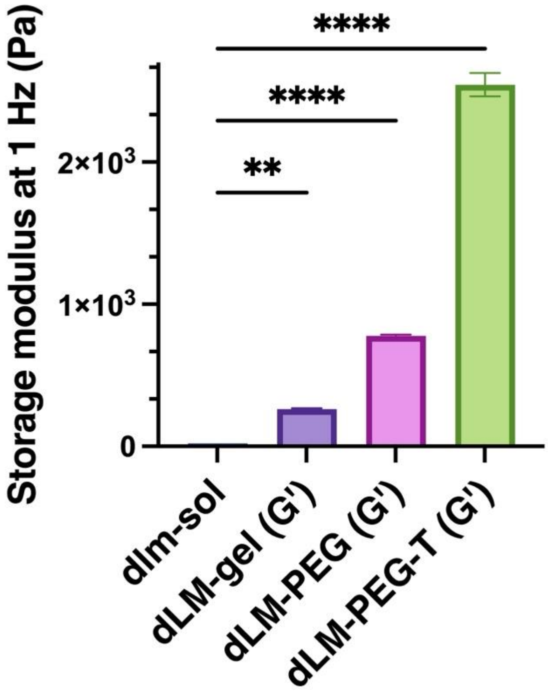

Figure S3. (B) The storage modulus of different crosslinked formulations and uncrosslinked formulations (dLM-sol) at a low 1 Hz frequency.

Figure S4. Channel formation with dLM-PEG-T after PVA dissolution (side view).

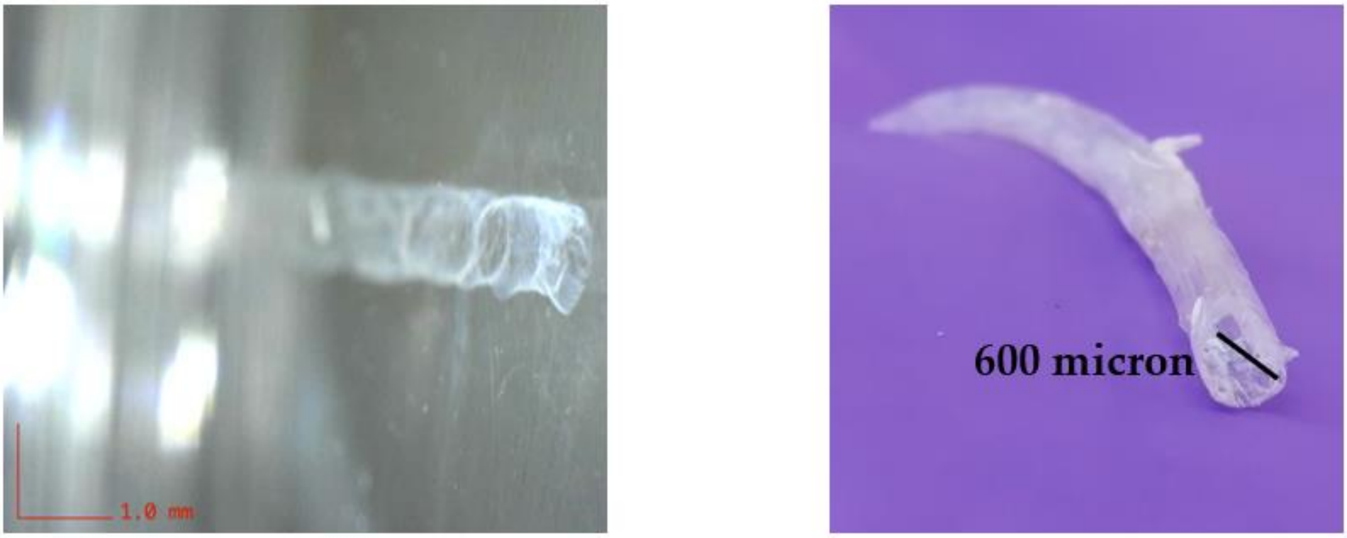

Figure S4. Micro-channel formed with 0.7 mm diameter (left) and after lyophilization (right).

Figure S5. Trilobular structure with dLM-PEG filled inside.

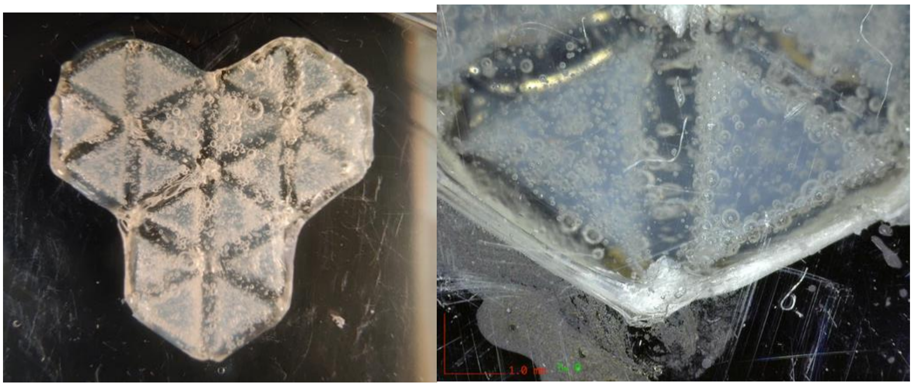

Figure S5. Trilobular hepatic structure formed by filling the dLM-PEG-T into the triangular pores of the 3D printed PVA construct.

Figure S6. Side view of the trilobular structure with dLM-PEG filled inside on day 0, day 7 and day 21.

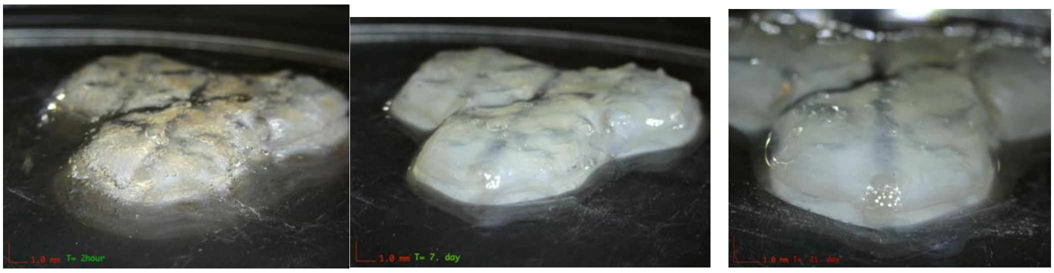

Figure S6. Day 0, day 7 and day 21 for the 3D structure with minute changes.

Figure S7. Long-term culture and degradation of the dLM-PEG-T structure.

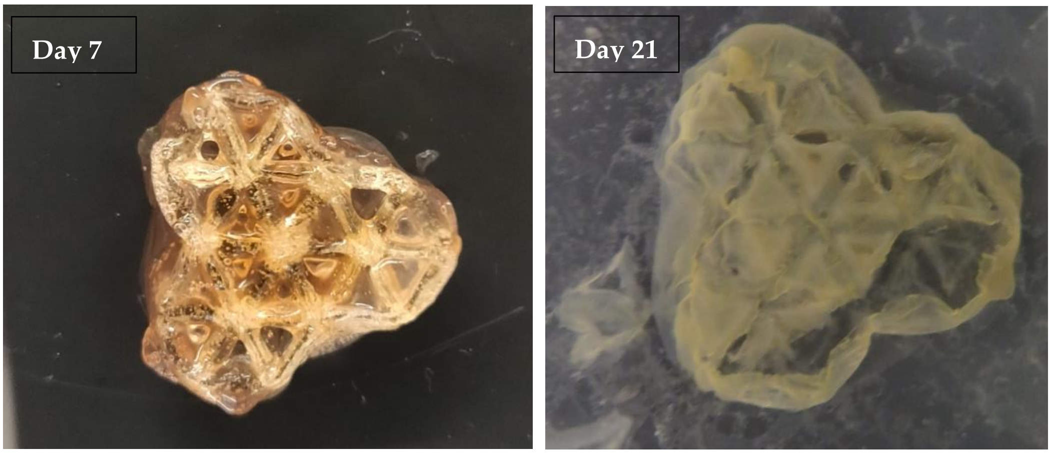

Figure S7. (A) dLM-PEG-T on day 7 (left) and day 21 (right).

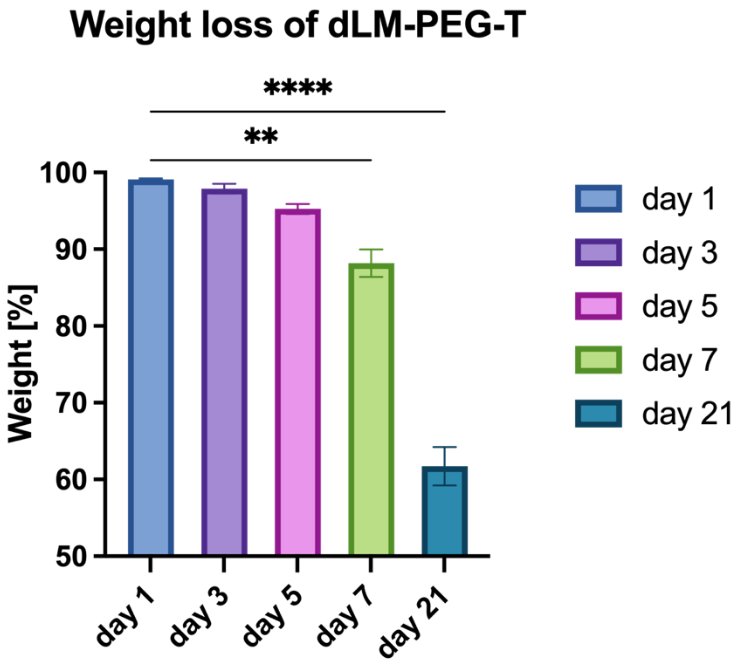

Figure S7. (B) Degradation kinetics of the dLM-PEG-T hydrogel relative to the hydrogel weighed immediately after crosslinking (n=3, ***$p < 0.01$ and **** $p < 0.0001$). Error bars represent the standard error of the mean.

Figure S8. The collagen control used as co-culture control with $HepG_2$ and NIH 3T3 cells.

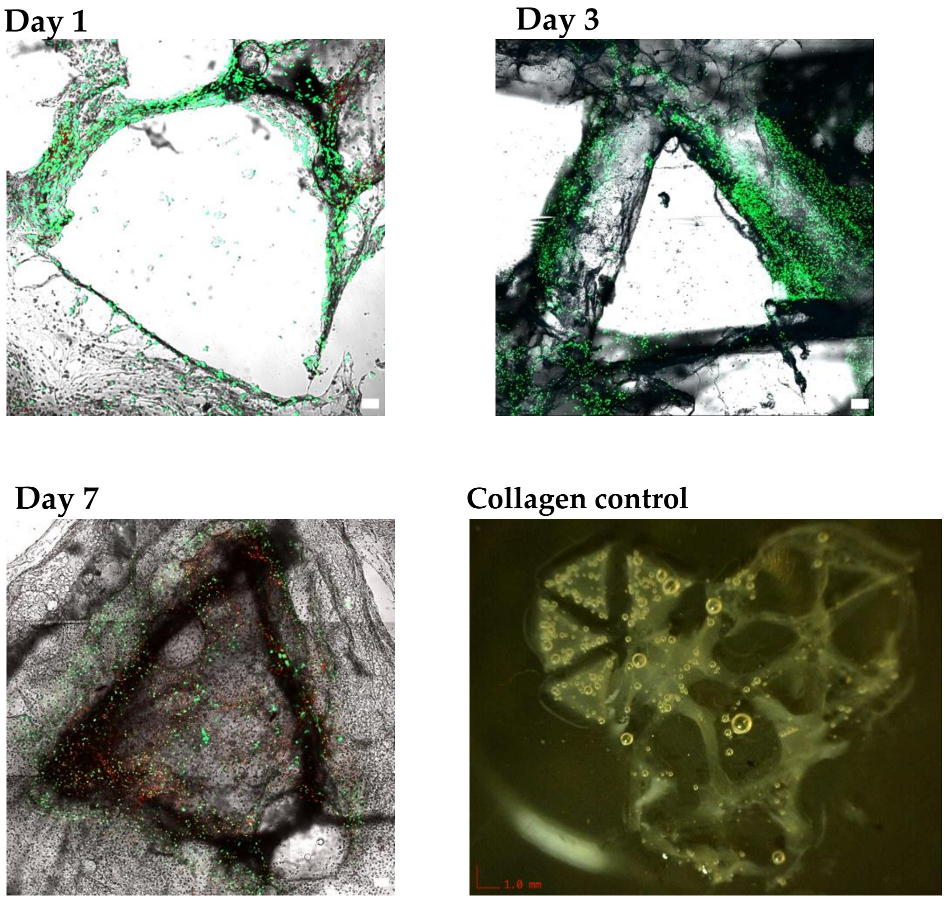

Figure S8. (A) Live (green) and dead (red) cells in the co-culture on day 1, day 3, and day 7 (scale bar 100 μm). The collagen control was used as a co-culture control with $HepG_2$ and NIH 3T3 cells.

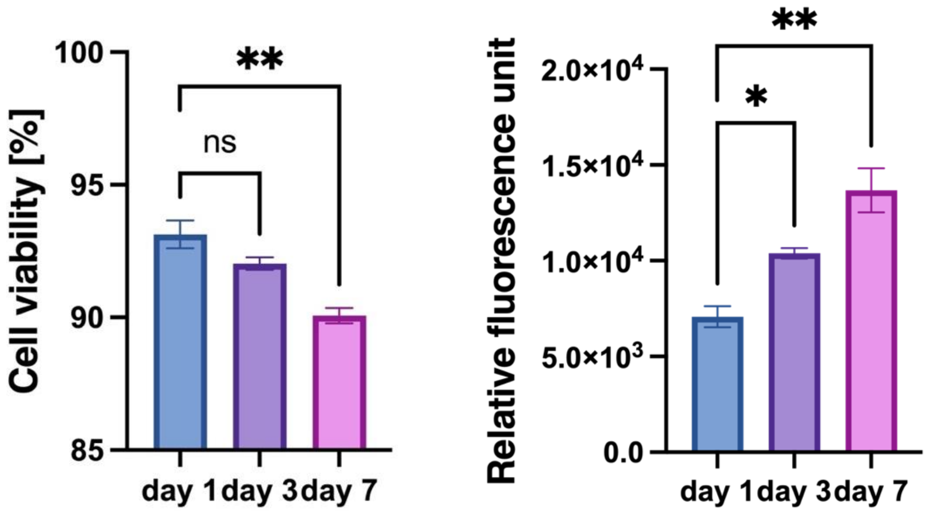

Figure S8. (B) Cell biocompatibility with collagen was analyzed with Live/dead assay on days 1,3 and 7 (scale bar 100 μm) and AlamarBlue™ assay at days 1, 3 and 7 (n=3, * $p < 0.05$ and ** $p < 0.01$). Error bars represent the standard error of the mean.

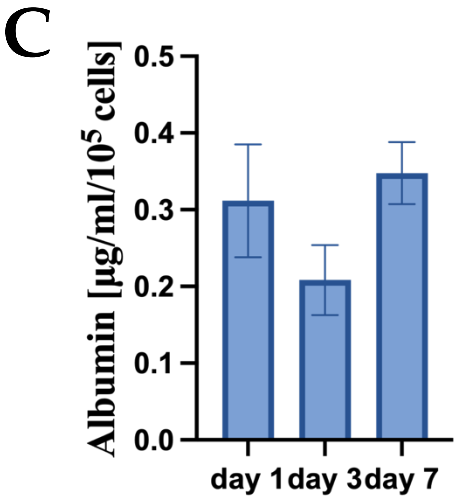

Figure S8. (C) Albumin secretion of the collagen control samples on days 1, 3 and 7 (n=3, * $p < 0.05$).

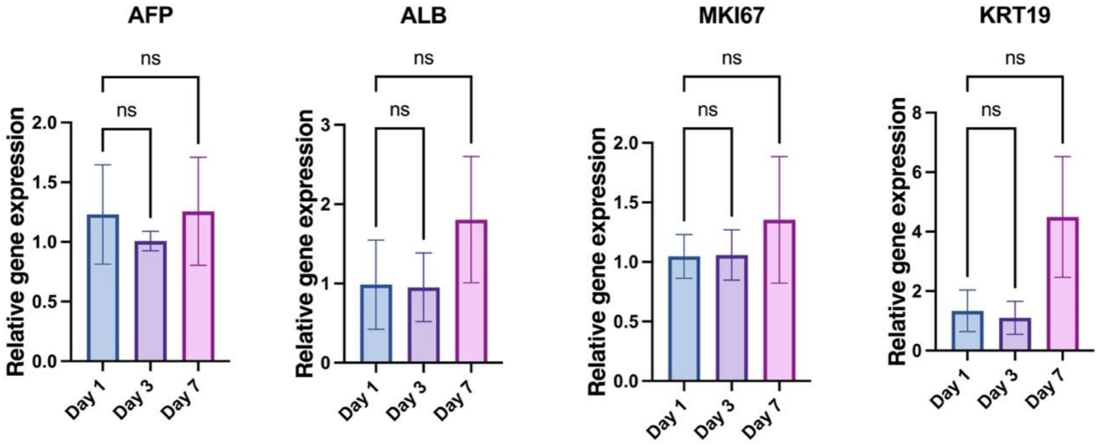

Figure S8. (D) Gene expression analysis of collagen co-culture on days 1, 3, and 7 (Error bars represent the standard error of the mean).

Figure S9. Migration study of HepG2 and NIH 3T3 cells seeded over 600 μm thick dLM-PEG-T hydrogel imaged at different time points.

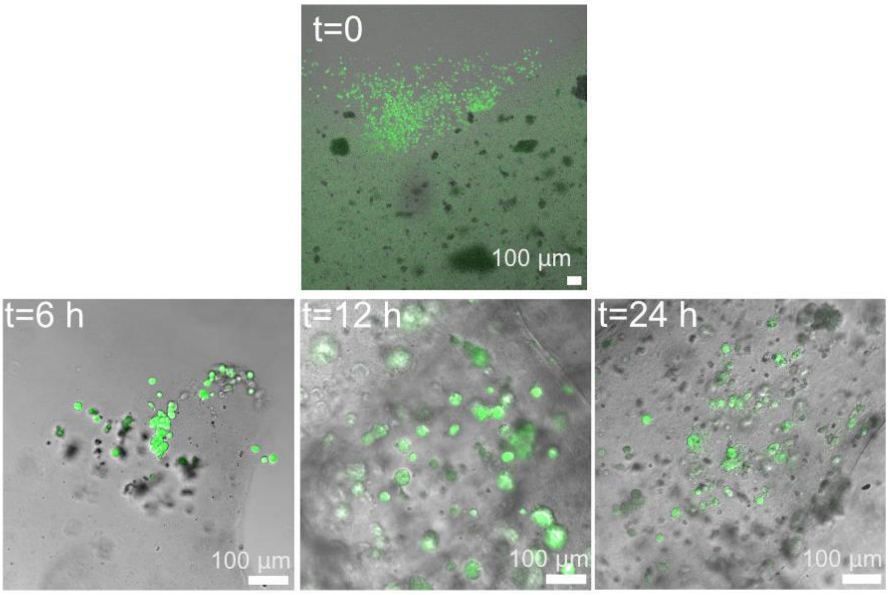

Figure S9. Live (green) cells after seeding the cells at t=0. Migration of live cells inside the dLM-PEG-T hydrogel at t=6, t=12 h, and t=24 h (scale bar 100 μm).

Figure S10. Urea secretion by the co-culture of $HepG_2$ and NIH 3T3 cells.

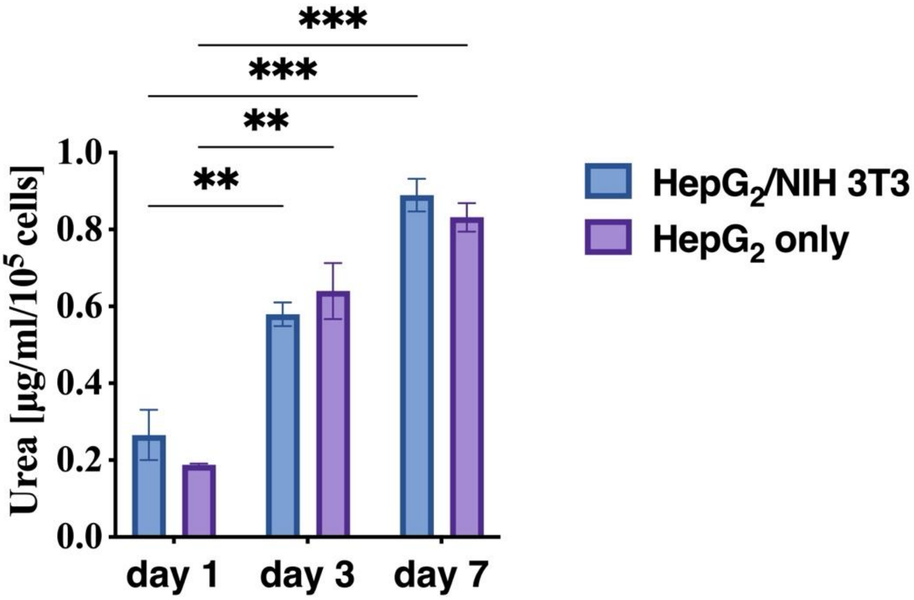

Figure S10. Urea expression (n=3, ** p < 0.01 and *** p < 0.001). Error bars represent the standard error of the mean.

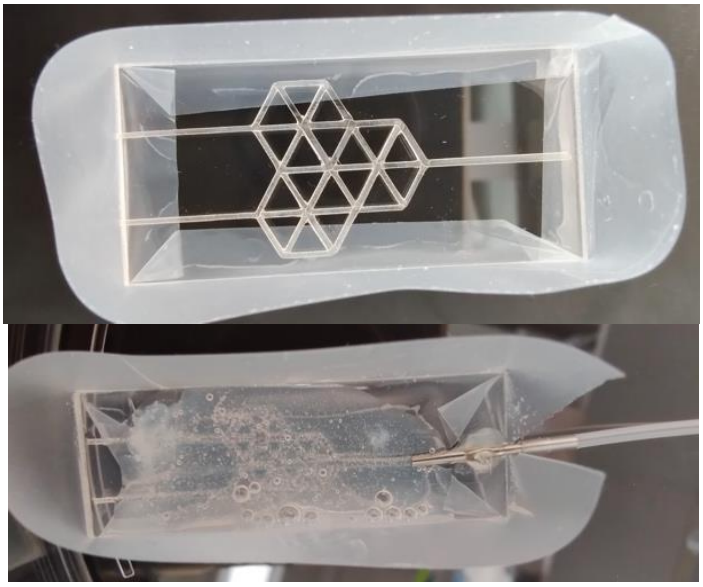

Figure S11. The liver lobule-on-chip concept for future studies. Top: empty chip with PVA. Bottom: PVA chip filled with dLM and solubilized to form channels.
